# Polymer-Coated Nanoarchitectonics of Titanium Dioxide Nanoparticles with Enhanced Antimicrobial Activity: Mechanistic Insights into Localized Particle–Membrane Interactions

**DOI:** 10.1021/acscentsci.6c00511

**Published:** 2026-06-18

**Authors:** Nizar B. Alsharif, Lucrezia Caselli, Anders Thapper, Emma Sparr, Martin Malmsten

**Affiliations:** † Department of Chemistry, 5193 Lund University, SE-22100 Lund, Sweden; ‡ Wallenberg Initiative Materials Science for Sustainability, Department of Chemistry, Lund University, SE-22100 Lund, Sweden; § Department of Chemistry, University of Florence, Via della Lastruccia 3, 50019 Sesto Fiorentino, Florence, Italy; ∥ Center for Colloid and Surface Science (CSGI), Via della Lastruccia 3, 50019 Sesto Fiorentino, Florence, Italy; ⊥ Department of Chemistry - Ångström, 8097 Uppsala University, SE-75120 Uppsala, Sweden; # Department of Pharmacy, University of Copenhagen, Copenhagen DK-2100, Denmark

## Abstract

While surface modifications are widely pursued to improve antimicrobial performance of photocatalytic nanoparticles (NPs), the particle–membrane interactions responsible for these effects remain underexplored. We address this gap by investigating the influence of coating TiO_2_ NPs with the cationic polymer poly­(2-(dimethylamino)­ethyl methacrylate) methyl chloride quaternary salt (qPDMAEMA). In contrast to bare TiO_2_, the coated NPs adsorb extensively to negatively charged bacteria and bacteria-like membranes, boosting membrane permeabilization upon UV illumination due to formation of reactive oxygen species (ROS). The qPDMAEMA coating was demonstrated not to interfere with ROS formation and to withstand UV illumination over time-scales sufficient for membrane binding and disruption. Such effects were highly localized near membrane-bound NPs, consistent with the short diffusion lengths of ROS (≈10 nm for hydroxyl radicals) and the formation of oxidative membrane ‘hot-spots’ in the corresponding vicinity of membrane regions where preferential (localized) NP binding occurs. Such preferential localization is demonstrated to occur at poles and nodes of *Escherichia coli* bacteria. Hypothesizing this to be driven by colocalization with anionic cardiolipin, studies with giant vesicles containing cardiolipin either uniformly distributed or present in segregated domains showed that the polymer-coated TiO_2_ NPs preferentially bind to cardiolipin-rich regions of the membrane. Together, these results expand on conventional studies of NP interactions with bacteria and bacteria-like membranes and demonstrate that localized interactions must be considered in studies of bacterial membrane interactions of photocatalytic NPs.

## Graphical abstract

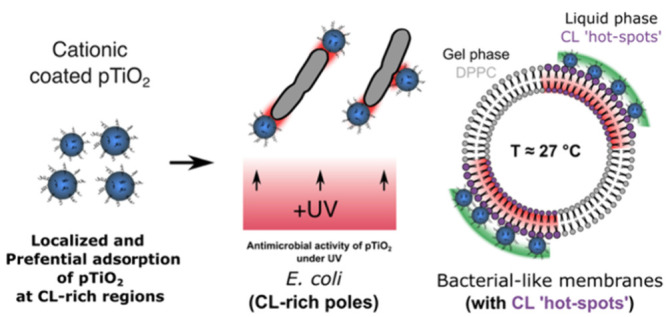

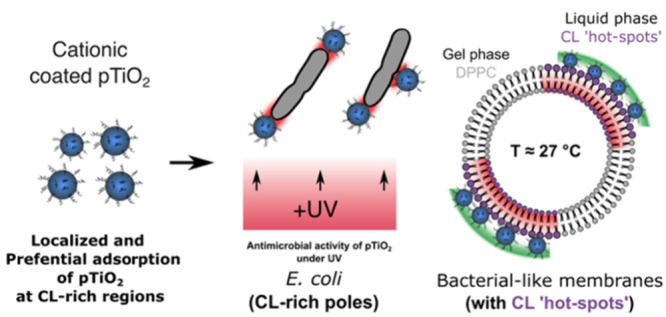

## Introduction

The emergence of antimicrobial resistance presents a major global health threat.^1^ In 2019, drug-resistant bacteria caused 1.27 million deaths and contributed to 4.95 million deaths.^2^ Water-borne infections significantly contribute to this, imposing profound suffering and a massive economic burden, as clean water remains unavailable to billions of people around the globe.^3,4^ For instance, contaminated water leads to 1.7 billion cases of diarrheal diseases alone each year, resulting in nearly 500 000 deaths.^5^ The decline in antibiotic development, coupled with the rise of antibiotic-resistant strains and antibiotic overuse/abuse, results in an urgent need for innovative approaches to counter antimicrobial resistance and waterborne pathogens alike.

Currently employed water disinfection methods have several drawbacks. For example, chlorination results in the formation of disinfection byproducts and shows limited effectiveness against key microorganisms such as *Giardia duodenalis* and *Cryptosporidium parvum*.^6,7^ Ozonation and UV treatment alone, on the other hand, are costly and do not maintain protection effect after treatment.^8,9^ In contrast, nanoparticles (NPs) with antimicrobial properties present a promising alternative due to their broad-spectrum and potent antimicrobial activity, as well as low-cost production.^10–14^ These materials exhibit antimicrobial properties through several mechanisms, including inhibition of metabolic pathways, membrane permeation, reactive oxygen species (ROS)-induced oxidative stress, as well as inhibition of bacterial enzymes and cellular functions via metal-ion release.^11,15^ Among these, photocatalytic NPs are gaining significant attention as antimicrobial agents due to their antimicrobial potency against a broad range of microorganisms including bacteria, viruses, and fungi.^16,17^

Despite numerous promising reports on potent antimicrobial effects of photocatalytic NPs, mechanistic aspects underlying these remain underexplored. This includes how NP properties affect their interactions with bacterial membranes, and how photocatalytic NPs can be designed for enhanced antimicrobial efficacy and selectivity between bacteria and human cells. Addressing this gap, we previously investigated coating photocatalytic TiO_2_ NPs with antimicrobial peptides (AMPs) and found such coated NPs to display boosted antimicrobial effects against both Gram-negative and Gram-positive bacteria, but at the same time showed low toxicity against human cells.^18,19^ These effects were demonstrated to correlate to preferential binding to bacterial membranes, which are rich in anionic phospholipids and void in cholesterol.^20–22^ Such selective association enables localization of the oxidative effects related to short ROS lifetime and corresponding diffusion lengths (≈10–100 nm) before consumption.^23^ Consequently, antimicrobial efficacy is expected to depend strongly on localized NP-membrane interactions, where preferential (high) NP binding to specific membrane regions may create oxidative ‘hot-spots’ of corresponding length scale which are selectively (particularly) prone to membrane destabilization. In these previous studies, however, the potential occurrence of localized binding and lysis sites of bacterial membranes was not addressed. Extending these previous studies, we here set out to address the issue of preferential binding and lysis localization.

To this end, we employed TiO_2_ NPs, which are one of the most extensively investigated photocatalytic NPs for water treatment and disinfection. While effective use of TiO_2_ NPs in water disinfection is limited by their large bandgap (requiring UV illumination),^13,24–27^ the large band gap makes TiO_2_ an ideal material for mechanistic studies of NP-membrane interactions since photocatalytic effects can be selectively triggered and deactivated as desired, allowing discrimination between photocatalytic and non-photocatalytic effects. In real-world applications, such photocatalytic nanomaterials need to be further engineered for use under visible light through strategies such as doping and heterojunction coupling. This reduces the band gap, which enhances collective ROS generation over the UV region, and decreases the charge recombination rate.^18^

For water disinfection, selectivity between bacteria and human cells is less critical than in therapeutic applications. Instead, boosting of antimicrobial effects is the primary concern since this relates not only to water safety but also to water processing infrastructure needed and the volume processing capacity.^28,29^ As demonstrated in our previous studies,^18,19^ cationic coating presents a simple yet effective approach to boosting antimicrobial effects, leveraging the major differences between bacterial and mammalian membranes. Thus, bacteria contain no cholesterol and are rich in anionic phospholipids, while human cells are rich in cholesterol and dominated by zwitterionic phospholipids.^20–22^ As demonstrated in our previous work, the combination of zwitterionic POPC (1-palmitoyl-2-oleoyl-*sn*-glycero-3-phosphocholine) and anionic POPG (1-palmitoyl-2-oleoyl-*sn*-glycero-3-phospho-(1’-rac-glycerol)) provides a simple and well-defined model system, which captures at least some features of the bacterial membrane regarding membrane interactions of NPs.^30,31^ While this model lacks the full complexity of native bacterial membranes, including components such as lipopolysaccharides, membrane proteins, and structural heterogeneity, cationically coated TiO_2_ NPs have previously been demonstrated to degrade bacterial lipid membranes and lipopolysaccharides alike.^18,19^ In addition, model lipid membranes provide a useful baseline for mechanistic studies of nanoparticle–membrane interactions. This, therefore, serves as a baseline system in the present study against which membrane domain features are contrasted.

With selectivity between bacteria and human cell membranes being of secondary importance in water purification, the use of expensive AMPs is unwarranted and economically unfeasible. With water purification applications in mind, this study instead focuses on surface modification of TiO_2_ NPs with the cationic polymer poly­(2-(dimethylamino)­ethyl methacrylate) methyl chloride quaternary salt (qPDMAEMA). The cationic and amphiphilic nature of qPDMAEMA is hypothesized to enable efficient coating of TiO_2_ NPs, enhance binding to bacterial membrane binding, promote membrane disruption, and provide better antimicrobial activity. Particle properties and membrane interactions were monitored with a battery of physicochemical approaches and correlated to bacterial membrane binding and antimicrobial effects. Furthermore, selective membrane-binding sites of pTiO_2_ in bacterial membranes were identified and correlated with localized membrane permeation on illumination. Together, the results demonstrate that pTiO_2_ binds selectively to cardiolipin-rich domains and that such localization needs to be considered when designing model systems in investigations of antimicrobial effects of (photocatalytic) NPs.

## Results and Discussion

### Characterization of NPs Properties

In aqueous solution, bare TiO_2_ NPs exhibit varying aggregation degrees at different pH conditions (Figure 1A). With an isoelectric point of 6.0–7.0,^32,33^ bare TiO_2_ NPs are positively charged in acidic media and become negatively charged at alkaline pH. At pH 7.4, TiO_2_ NPs carry little overall charge and hence undergo heavy aggregation, as evidenced by the increase in hydrodynamic size measured by DLS (Figure 1A) and confirmed by cryoTEM imaging (Figure S1, Supporting Information). To prevent this, TiO_2_ NPs were coated with qPDMAEMA at different concentrations (Figure 1B). At low qPDMAEMA concentration, coated TiO_2_ NPs had low zeta potential and underwent heavy aggregation. At 300 mg/g, however, qPDMAEMA adsorption to TiO_2_ NPs resulted in a highly positive zeta potential and formation of nanoparticles in the 30–100 nm size range. With the primary TiO_2_ NPs being 8 nm,^18^ these results indicate that each pTiO_2_ NP consists of a cluster of multiple TiO_2_ NPs, as also demonstrated by cryoTEM (Figure 1C). Higher qPDMAEMA concentrations did not result in any significant changes in zeta potential, indicating adsorption saturation. Illustrating the stability of the surface coatings, neither the particle size nor the zeta potential of pTiO_2_ at qPDMAEMA concentrations of 300, 1000, and 3000 mg/g changed significantly over 13 days (Figure S2, Supporting Information). The saturation of TiO_2_ NPs with qPDMAEMA was assessed by liquid-state ^1^H NMR (Figure S3, Supporting Information), which detects signals mainly from unbound qPDMAEMA. This was done by observing the peak at ∼3.2 ppm, characteristic of quaternary ammonium protons [−N^+^(CH_3_)_3_], as reported elsewhere in the literature.^34–36^ To identify the polymer saturation point, spectra of pTiO_2_ samples (fixed TiO_2_ amount, varying qPDMAEMA concentrations) were compared with those of qPDMAEMA only solutions at corresponding polymer concentrations. At 300 mg/g, the intensity of qPDMAEMA peak was significantly attenuated, compared to spectra of qPDMAEMA only. This indicates that, at 300 mg/g, most of the polymer was adsorbed onto TiO_2_. At 1000 mg/g, on the other hand, excess polymer remained free in the solution, resulting in similar peak intensities for pTiO_2_ and qPDMAEMA only samples. EPR spectra (Figure 1D) of UV-irradiated TiO_2_ and pTiO_2_ NPs incubated with DMPO showed the characteristic four-line signal of the DMPO–·OH spin adduct, closely matching simulated spectra (Figure 1E) and consistent with the literature.^37,38^ This confirms the predominant generation of hydroxyl radicals (·OH). In addition, control (individual) EPR spectra of qPDMAEMA and DMPO showed no detectable EPR signals (Figure 1E). The combination of DMPO + TiO_2_ NPs in darkness exhibited negligible signal, confirming that the observed DMPO–·OH signal arises following UV illumination. In the presence of pTiO_2_, the ·OH radical formation showed a time-dependent increase in signal intensity, which approached a near plateau after 27 min of UV illumination, suggesting that DMPO–·OH formation reached a steady state (Figure 1F).

**Figure 1:**
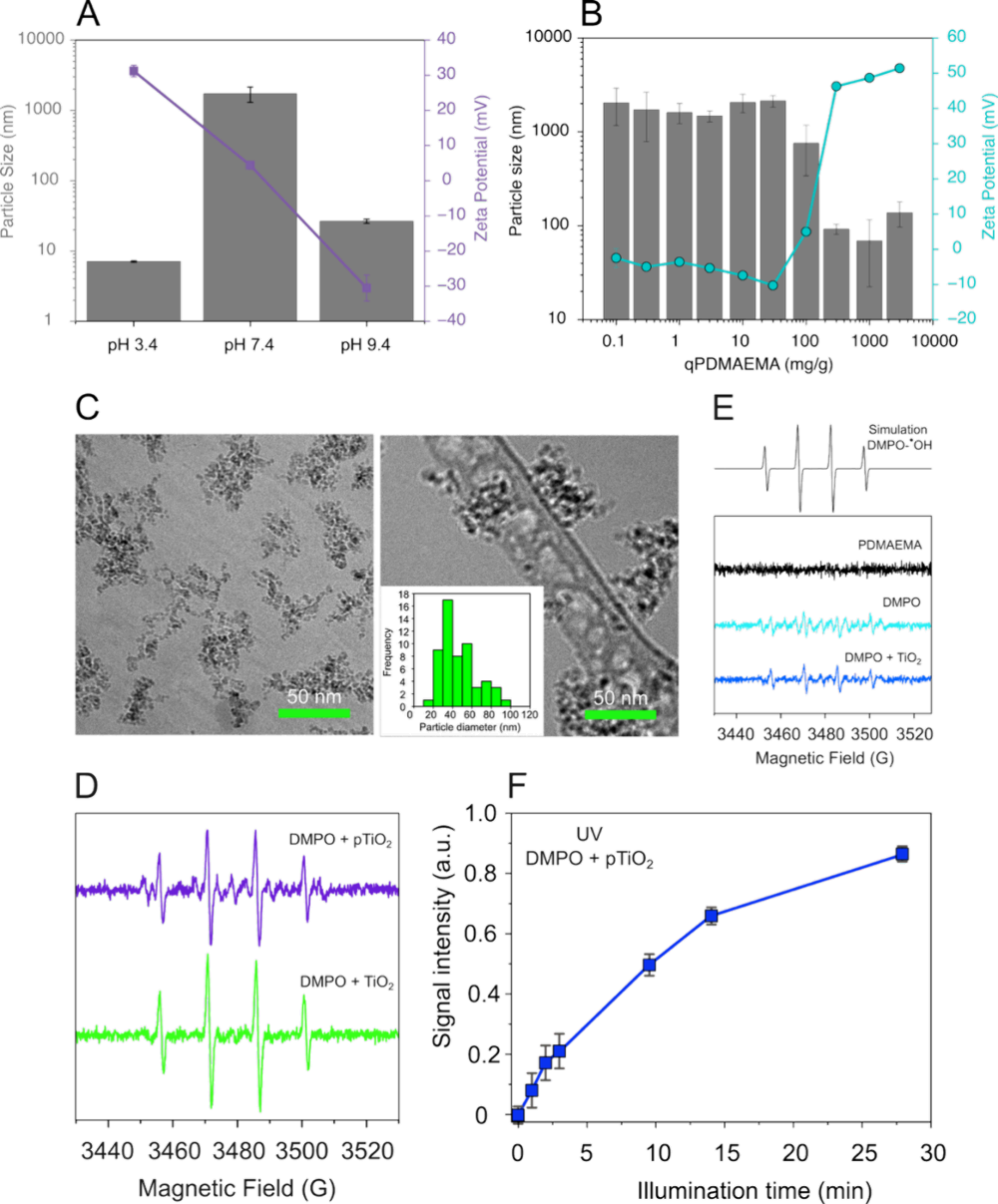
Characterization of TiO_2_ NPs. (A) Hydrodynamic diameters and zeta potential values of bare TiO_2_ NPs (50 mg/L) at various pH values. (B) Hydrodynamic diameters and zeta potential values of pTiO_2_ (50 mg/L TiO_2_) at various qPDMAEMA concentrations (mg qPDMAEMA/g TiO_2_) at pH 7.4. (C) Representative cryoTEM images of pTiO_2_ (300 mg/g, 50 mg/L TiO_2_) in Tris buffer. (D) EPR spectra showing the DMPO-·OH adduct signal after 10 min of UV illumination in the presence of bare TiO_2_ NPs or pTiO_2_. (E) Simulation results for the DMPO-·OH signal, along with individual EPR spectra of free qPDMAEMA, DMPO trapping agent, and DMPO + TiO_2_ NPs, obtained in darkness under similar conditions. (F) Quantification of DMPO-·OH adduct formation over 27 min of UV illumination in the presence of pTiO_2_. The EPR samples were measured in MQ water at pH ≈7.0.

### ROS-Induced Lipid Oxidation

In the absence and presence of radical scavengers, UV-induced lipid oxidation was further assessed by monitoring fluorescence spectra of C_11_-BODIPY-labeled vesicles. When no radical scavengers are present, the presence of pTiO_2_ led to a sharp increase in the oxidation rates of C_11_-BODIPY (Figure 2A), compared to bare TiO_2_ and particle-free sample. In addition, the presence of SOD (·O_2_^–^ scavenger) was found to have a minor impact on the oxidation rates. In comparison, introduction of d-mannitol (d-Mann, ·OH scavenger) led to a more sizable reduction in the C_11_-BODIPY oxidation rate, especially for pTiO_2_ (Figure 2A), indicating that ·OH radicals play a major role in C_11_-BODIPY oxidation, in agreement with the EPR results. The final oxidation levels over 40 min reflect the trends in the oxidation rates, i.e., higher oxidation level in the presence of pTiO_2_, and notable reduction in the oxidation level occurs in the presence of d-mannitol (Figure S4, Supporting Information).

**Figure 2:**
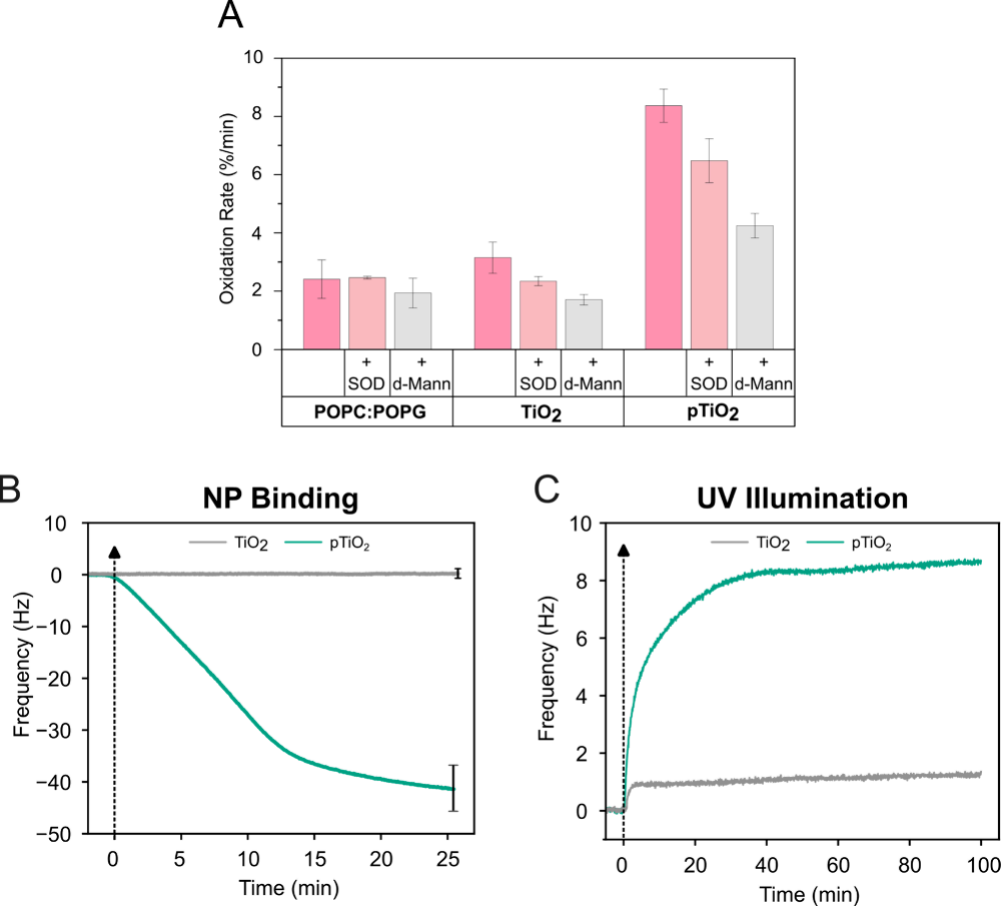
(A) Oxidation rates of C_11_-BODIPY-labeled vesicles incubated with bare TiO_2_ or pTiO_2_ under UV illumination for 40 min, as well as a particle-free (control) sample. Additional experiments were separately performed in the absence or presence of radical scavengers: 50 U/mL SOD or 500 mM d-mannitol (d-Mann). (B) QCM-d profile showing relative frequency changes during bilayer interaction with bare TiO_2_ NPs (20 mg/L) and pTiO_2_ (300 mg/g, 20 mg/L TiO_2_). (C) QCM-d profile showing oxidation-induced frequency changes during UV treatment of NP-loaded lipid bilayers. In (B), the arrow represents the onset of NP injection, and frequencies are relative to those prior to particle deposition. In (C), the arrow indicates the onset of UV illumination, and reported frequencies are relative to those after particle deposition in (B).

### Lipid Bilayer Interactions

To obtain information on how lipid oxidation, monitored above by EPR and C_11_-BODIPY, influences phospholipid membrane stability, QCM-d measurements were next performed. After bilayer formation and Tris buffer rinsing (Figure S5, Supporting Information), addition of positively charged pTiO_2_ (300 mg/g) led to a significant decrease in frequency (Figure 2B), indicating substantial NP binding, with some trapped water between NPs in the adsorbed layer likely contributing to the frequency change. In contrast, weakly negatively charged bare TiO_2_ showed no affinity toward the bilayer, in agreement with previous findings.^18^ On UV illumination, a decrease in the absolute frequency values was observed for pTiO_2_, which can be attributed to a loss of lipid and/or water from the adsorbed layer (Figure 2C). QCM-d profiles of qPDMAEMA binding to the bilayer show strong and irreversible adsorption, demonstrated by steady frequency values following prolonged buffer rinsing (Figure S6, Supporting Information). Furthermore, UV treatment of the bilayer in the absence of particles resulted in minor and reversible changes in the frequency/dissipation (Figure S7, Supporting Information). Full QCM-d profiles showing the interaction of the bilayer with bare TiO_2_ NPs, pTiO_2_ (300 mg/g), and pTiO_2_ (1000 mg/g) before, during, and after UV treatment are compared in Figure S8, Supporting Information. Compared with pTiO_2_ (300 mg/g), the interaction of the bilayer with pTiO_2_ (1000 mg/g) resulted in smaller changes in frequency and dissipation values, where fewer particles adsorb on the bilayer due to the higher presence of free qPDMAEMA, as seen in the NMR results. Hence, only pTiO_2_ (300 mg/g) was used in further experiments.

### Vesicle Permeabilization

To elucidate the impact of pTiO_2_ on membrane integrity, an assay employing Liss Rhod PE-labeled POPC:POPG GUVs (red) and the water-soluble dye Alexa Fluor 488 (green) was performed. As illustrated schematically in Figure 3A, intact GUV membranes are impermeable to Alexa Fluor 488, while membrane permeation occurs when sufficient concentrations of membrane-permeabilizing agents (e.g., qPDMAEMA or NPs) accumulate on the vesicle surface. As shown in Figure 3B, no significant changes in GUV permeability were observed at low free qPDMAEMA concentrations (≤0.3 mg/L), with no detectable fluorescence inside the vesicle lumen. In contrast, within the concentration range of 0.5–1.0 mg/L, qPDMAEMA binding was sufficient to induce membrane permeabilization in a concentration-dependent manner, as evidenced by increasing Alexa Fluor 488 fluorescence within the vesicles. The fraction of permeabilized GUVs at different polymer concentrations was quantitatively analyzed, as shown in Figure 3C, with more permeation observed at higher polymer concentrations.

**Figure 3:**
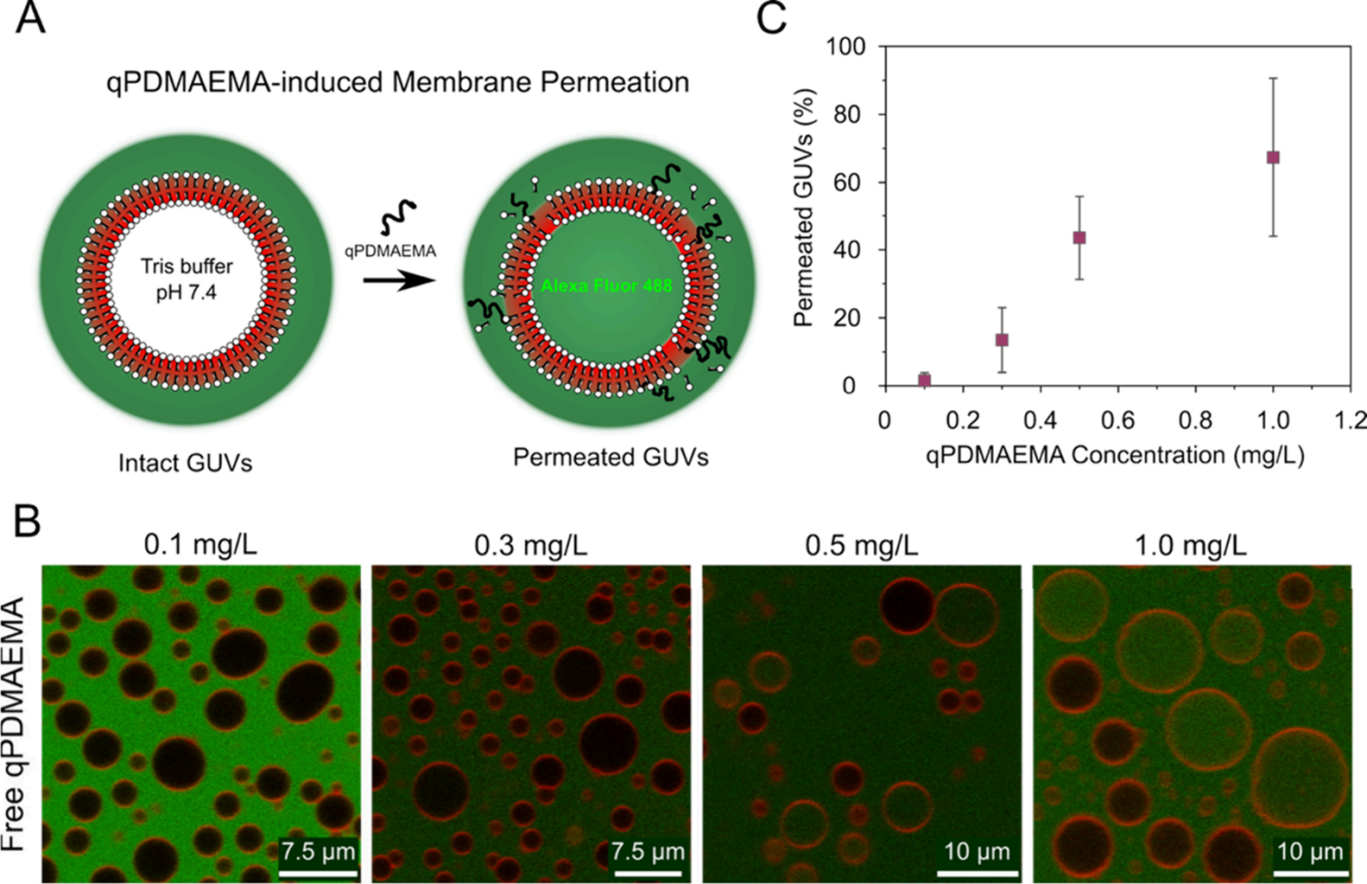
(A) Schematic illustration of red-fluorescing Liss Rhod PE-labeled POPC:POPG GUVs, which remain impermeable to Alexa Fluor 488 in the absence of qPDMAEMA (green dye only outside the GUVs). Upon addition of polymer, membrane permeabilization allows Alexa Fluor 488 to fill also the inside lumen of the GUVs. (B) Representative confocal microscopy images showing the effect of increasing concentrations of free qPDMAEMA (0.1–1.0 mg/L) on GUVs membrane permeability: (i) no discernible membrane permeation at low concentrations (0.1–0.3 mg/L) and (ii) concentration-dependent membrane permeation at higher concentrations (0.5–1.0 mg/L), indicated by Alexa Fluor 488 fluorescence within the vesicle lumen. (C) Quantitative analysis of GUVs permeation at different polymer concentrations, presented as the average fraction of permeated vesicles from 10 images per concentration.

In contrast to observations with free qPDMAEMA, the introduction of bare TiO_2_ NPs did not induce any detectable GUV permeability, even after UV irradiation (Figure S9, Supporting Information). Similarly, the addition of pTiO_2_ in darkness did not affect GUVs permeability (Figure 4A, 0 min UV). On UV irradiation, however, membrane integrity was significantly compromised over time, with extensive membrane permeation of Alexa Fluor 488 observed (Figure 4A, 120 min UV). Furthermore, the presence of pTiO_2_ particles on the membrane surface was directly visualized in bright-field microscopy images (Figure 4B) at sites of localized membrane permeabilization, as identified by compromised membrane integrity in the red fluorescence channel. These findings are all consistent with the C_11_-BODIPY (Figure 2A) and QCM-d (Figure 2B–C) results on lipid oxidation and membrane destabilization, respectively.

**Figure 4:**
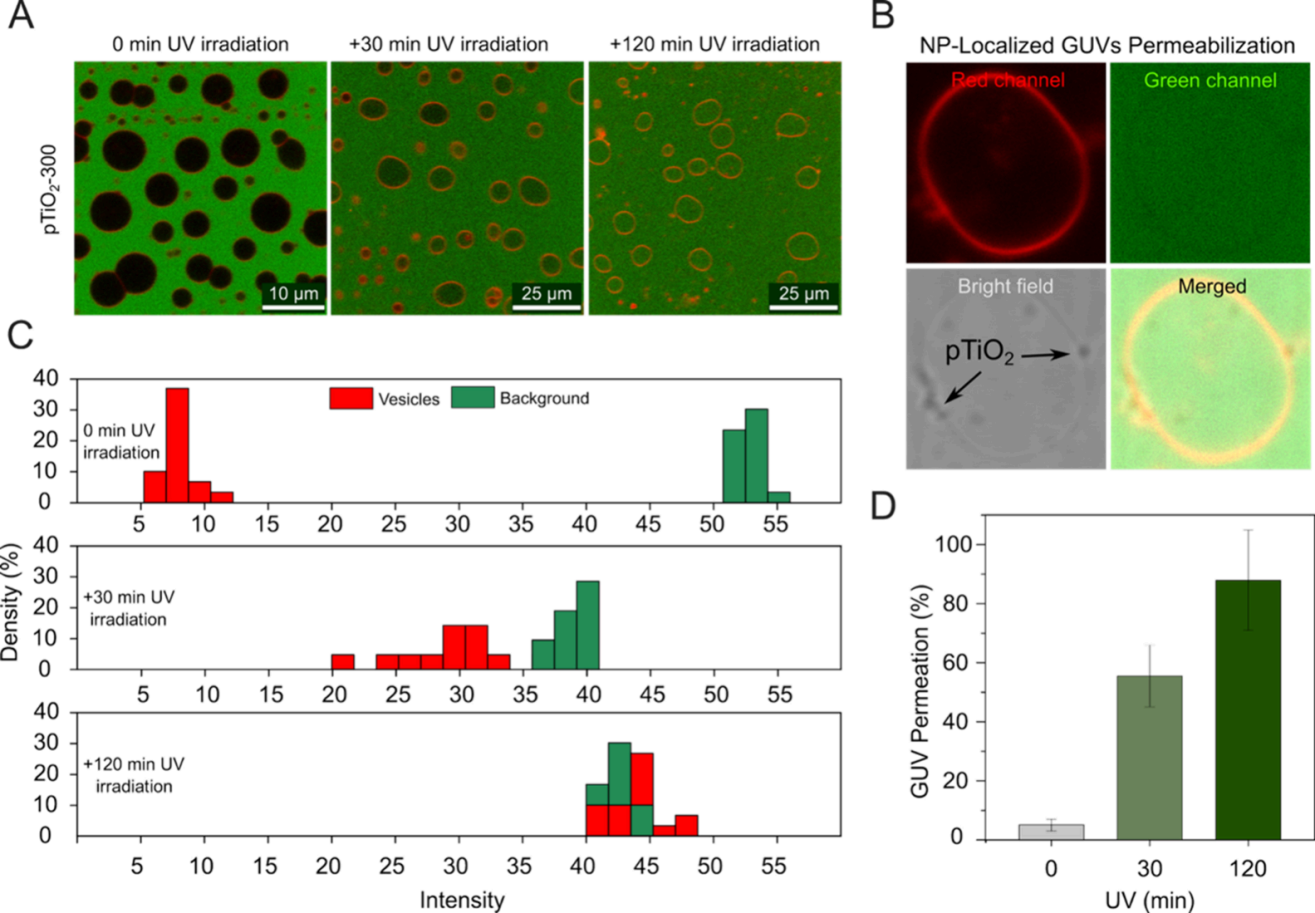
(A) Confocal microscopy images of Liss Rhod PE-labeled POPC:POPG GUVs incubated with 1 mg/L pTiO_2_ in Tris buffer (1 mM, pH 7.4), showing progressive membrane permeabilization during UV irradiation. (B) Representative confocal images demonstrating localized pTiO_2_-induced membrane permeation at nanoparticle adsorption sites after 120 min of UV exposure. (C) Fluorescence intensity analysis of Alexa Fluor 488 inside (red bars) and outside (green bars) GUVs shown in (A) before irradiation (0 min), during irradiation (+30 min), and after prolonged UV exposure (+120 min). (D) Quantitative analysis of GUV permeation in (A) as a function of UV irradiation time. Values represent the average fraction of permeated vesicles from 10 images per time point (total of 740 GUVs analyzed).

The extent of membrane permeation by pTiO_2_ under UV irradiation in Figure 4A was quantified by analyzing the fluorescence intensity of Alexa Fluor 488 within and outside vesicles, based on confocal images taken during the 2-h irradiation period (Figure 4C). Prior to UV illumination, the GUVs were largely impermeable with low fluorescence inside and high fluorescence outside the vesicles. After 30 min of illumination, membrane integrity was compromised, as indicated by the increased fluorescence inside the vesicles (Figure 4C). After 120 min of illumination, full vesicle permeation was observed with equal fluorescence intensity inside and outside the vesicles (Figure 4C). Quantitatively, the fraction of GUVs in Figure 4A exhibiting lost membrane integrity increased with illumination time (Figure 4D), where pTiO_2_ induced permeation in 88 ± 16% of GUVs after 120 min of UV illumination.

To monitor the spatial distribution of ROS-induced oxidation, C_11_-BODIPY oxidation was examined by confocal microscopy for C_11_-BODIPY-labeled GUVs (Figure 5A). Green- and red-channel images of the GUVs incubated with 20 mg/L pTiO_2_ were acquired before and after UV treatment. Prior to UV illumination (Figure 5B), C_11_-BODIPY emitted red fluorescence, while no detectable green fluorescence was observed, indicating that no oxidation had occurred. After UV illumination, significant green fluorescence (oxidized C_11_-BODIPY) was observed, while the red fluorescence decreased below the detection limit, reflecting the dominance of oxidized C_11_-BODIPY. Consistent with the hydrophobicity of C_11_-BODIPY, oxidation was observed only within the membrane and neither in the vesicle interior lumen nor in the bulk solution outside the vesicles.

**Figure 5:**
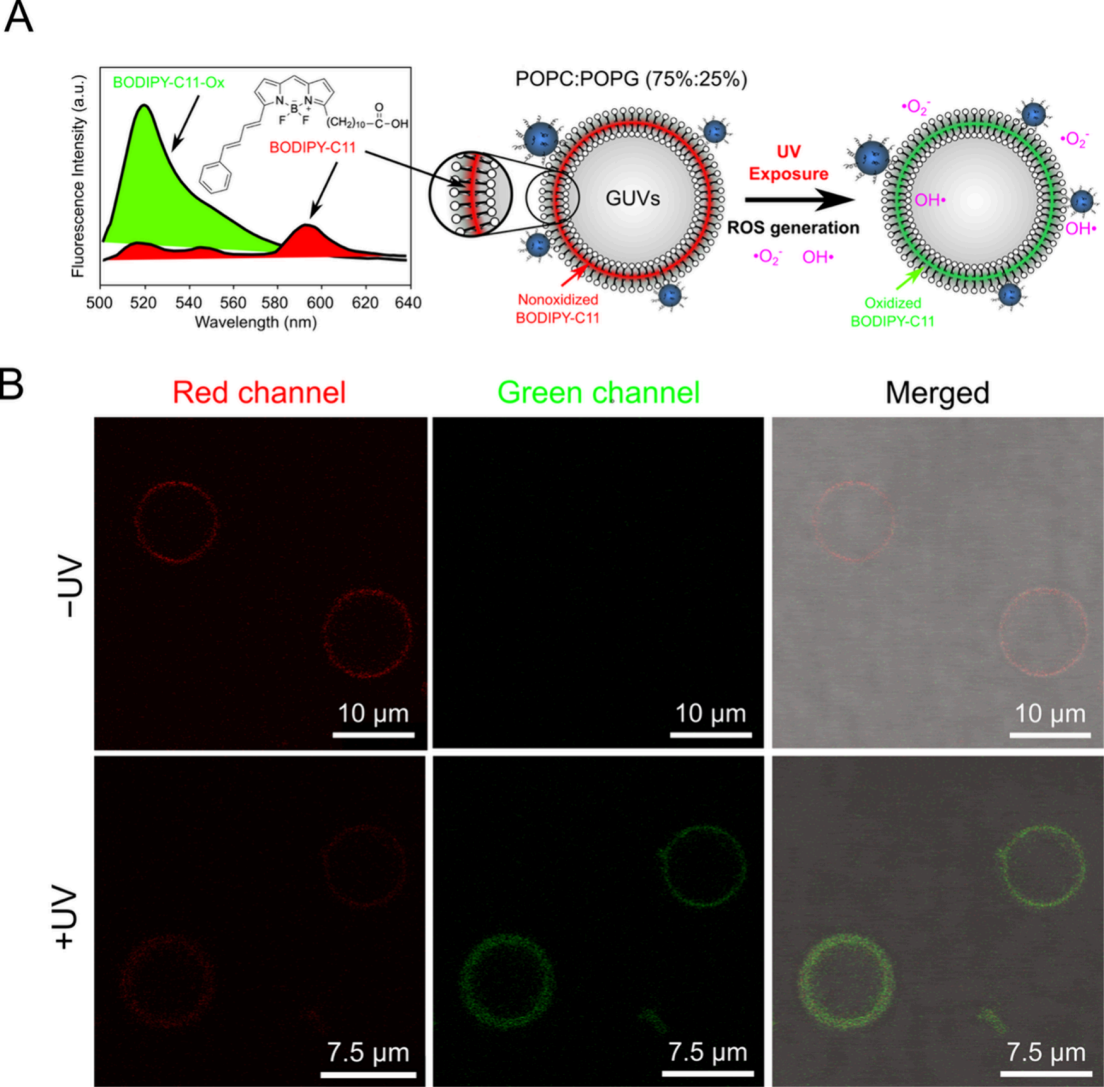
C_11_-BODIPY assay coupled with confocal microscopy. (A) Schematic illustration of the use of confocal microscopy to visualize membrane-localized C_11_-BODIPY oxidation, showing that UV illumination induces a spectral shift in membrane-bound C_11_-BODIPY, which can be spatially observed by confocal microscopy. (B) Confocal microscopy images of C_11_-BODIPY-labeled GUVs incubated with pTiO_2_ (20 mg/L), before and after 120 min of UV illumination. Shown are green and red channels, as well as merged overlay.

### Antimicrobial Effects

To evaluate whether the effects observed in model lipid membranes translate to bacteria, the antimicrobial effects of bare TiO_2_ and pTiO_2_ NPs were next assessed using the LIVE/DEAD assay. Red and green staining of Gram-negative *E. coli* bacteria at pH 7.4 was imaged by confocal microscopy to distinguish intact (green) from damaged (red) cellular membranes (Figure 6A). After UV illumination, control (particle-free) samples showed modest bacterial killing (29 ± 10% dead bacteria), attributed to UV absorption. The presence of bare TiO_2_ NPs did not enhance antimicrobial effects after UV treatment (20 ± 2% dead bacteria). In the dark, pTiO_2_ (5 mg/L) showed modest antimicrobial activity, similar to that of control, bare TiO_2_ NPs (Figure 6A), and free qPDMAEMA (Figure S10, Supporting Information). Upon UV illumination, however, pTiO_2_ showed a pronounced increase in antimicrobial activity, resulting in 76 ± 8% dead bacteria (Figure 6B). Statistical analysis revealed highly significant differences among all six treatment groups (one-way ANOVA, *F*(5, 114) = 575.3, *p* < 0.0001). Planned Welch’s *t*-tests further demonstrated that UV-activated pTiO_2_ induced significantly greater bacterial killing than UV-treated control samples (*t*(35) = 16.3, *p* < 0.0001), bare TiO_2_ under UV illumination (*t*(34) = 20.7, *p* < 0.0001), and nonirradiated pTiO_2_ (*t*(21) = 32.1, *p* < 0.0001). These findings confirm that qPDMAEMA functionalization markedly enhances TiO_2_-mediated antimicrobial activity under UV activation while maintaining negligible dark toxicity.

**Figure 6:**
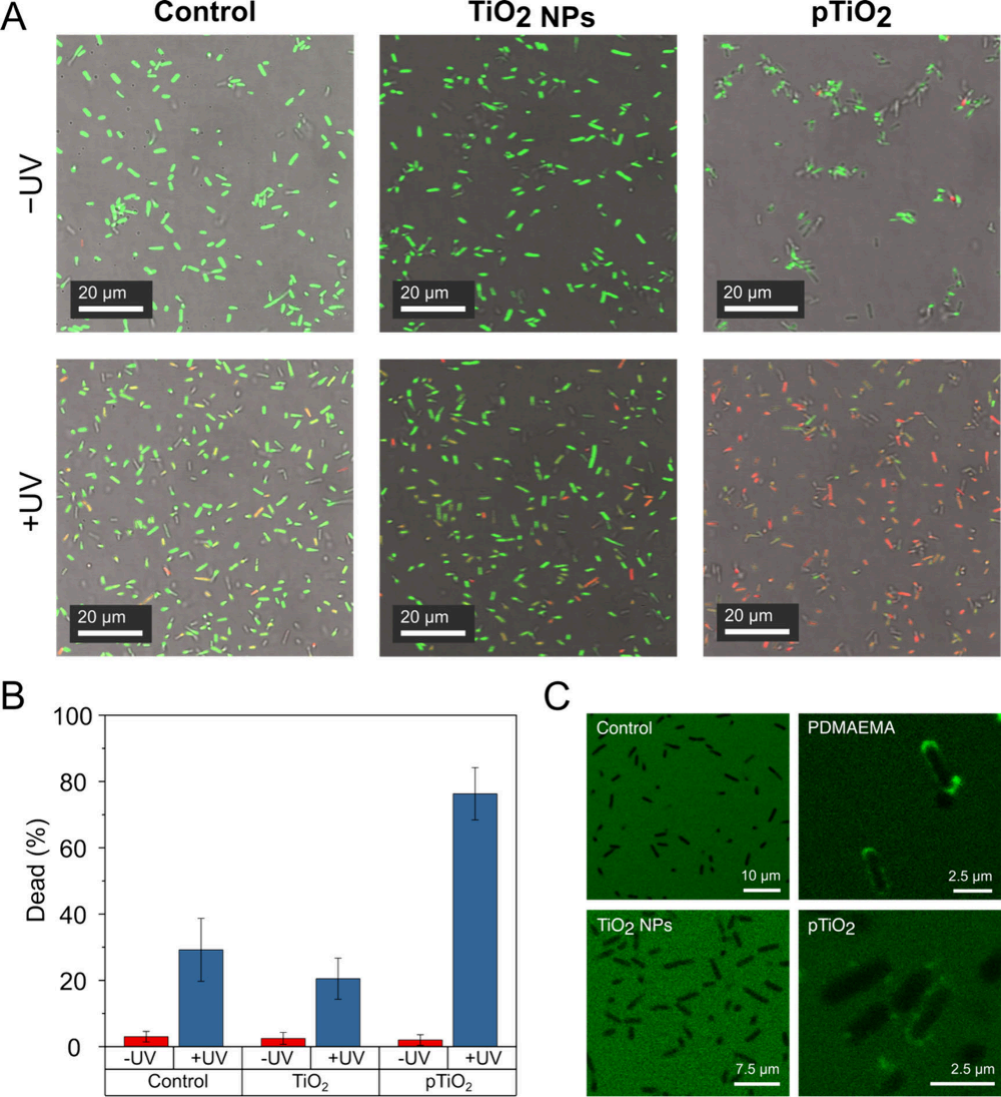
(A) Confocal microscopy images with bright-field overlay for the LIVE/DEAD assay in Tris buffer. Shown are representative images for control (particle-free) *E. coli* samples (6 × 10^8^ CFU/mL), as well as for bacteria incubated with bare TiO_2_ NPs and pTiO_2_ (5 mg/L TiO_2_) before (−UV) and after (+UV) UV illumination. (B) Quantitative analysis of confocal microscopy images, showing the percentage of dead *E. coli* bacteria (Dead%). (C) Confocal microscopy images of *E. coli* suspended in Tris buffer containing dispersed Alexa Fluor 488 (green) after incubation with qPDMAEMA (3 mg/L), bare TiO_2_ NPs (10 mg/L), or pTiO_2_ (10 mg/L).

### Localized pTiO_2_ Binding

Following preaddition of either free qPDMAEMA or pTiO_2_, the subsequently added Alexa Fluor 488 localized preferentially at the poles of intact-membrane *E. coli* or, to a lesser extent, appeared in patches along the lateral wall (Figure 6C). Thus, the nonuniform distribution of Alexa Fluor 488 at the *E. coli* bacteria membranes suggests a corresponding nonuniform distribution of pTiO_2_ (and qPDMAEMA). This pattern may, in turn, arise from an uneven distribution of anionic lipids or lipopolysaccharides within the membrane.^39^ One prime candidate for this is cardiolipin, which has previously been reported to localize preferentially in poles and nodes (elongation sites) of Gram-negative bacteria.^40–42^ To investigate whether membranes with biologically relevant concentrations of cardiolipin can preferentially localize pTiO_2_ within a multicomponent bacteria-like membrane, 18:1 cardiolipin (CL) was incorporated in 0.5 mol % Liss Rhod PE-labeled GUVs with a lipid composition [DPPC:DOPC:CL (43.5:43.5:12.5 mol %)] selected to exhibit domain segregation at room temperature, and a single uniform phase at elevated temperatures (>40 °C) (Figure 7A). At elevated temperature, single-phase GUVs are characterized by uniform CL and Liss Rhod PE distribution (Figure 7B, single-phase GUVs panel). At room temperature, however, CL and Liss Rhod PE preferentially partition into the fluid DOPC-rich domains, whereas the DPPC-rich gel-phase domains appear dark (Figure 7B, two-phase GUVs panel). In the absence of pTiO_2_, Alexa Fluor 488 showed no significant membrane association at any temperature (Figure 7B). Addition of pTiO_2_ to single-phase GUVs (*T* = 45 °C) resulted in uniform binding of anionic Alexa Fluor 488 (green) over the membrane (Figure 7C, single-phase GUVs panel), indicating uniform pTiO_2_ membrane binding. In contrast, when pTiO_2_ was introduced to segregated GUVs (*T* = 27 °C), Alexa Fluor 488 distribution mirrored the Liss Rhod PE (red) distribution, suggesting preferential pTiO_2_ localization in the fluid membrane domains (Figure 7C, two-phase GUVs panel). Similar behavior was observed after adding free qPDMAEMA (Figure S11, Supporting Information). Further supporting this, QCM-d results showed qPDMAEMA to bind extensively to DPPC:DOPC:CL membranes but not to DPPC:DOPC membranes lacking CL (Figure 7D). In addition, negatively charged Alexa Fluor 488 does not bind to bacteria-like POPC:POPG membranes, unless these are preloaded with qPDMAEMA (Figure 7E). Together, these results demonstrate that CL-rich domains can indeed play a major role in mediating localized binding of qPDMAEMA and pTiO_2_.

**Figure 7:**
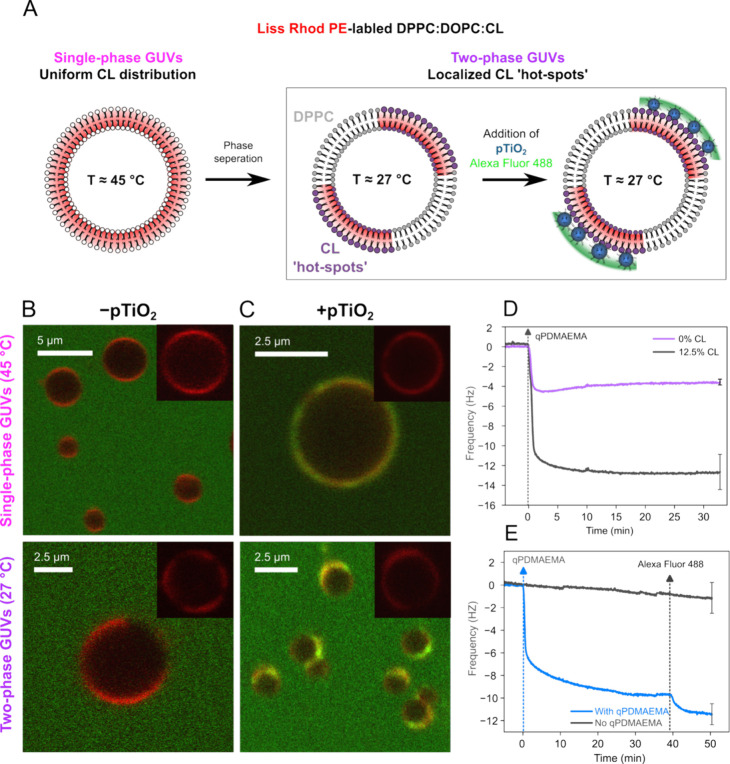
(A) Schematic illustration of temperature-induced phase separation of Liss Rhod PE-labeled DPPC:DOPC:CL GUVs, as well as subsequent localization of pTiO_2_ and Alexa Fluor 488, showing that pTiO_2_ NPs bind preferentially to CL-rich domains, and that Alexa Fluor 488 binds preferentially to the membrane-bound NPs. (B) Confocal microscopy images of single-phase GUVs (imaged at 45 °C) and phase-separated GUVs (imaged at 27 °C), dispersed in Alexa Fluor 488. (C) Fluorescence mapping of CL-mediated pTiO_2_ (20 mg/L) localization onto the GUVs. Upon cooling to 25 °C, phase separation into CL-rich (red) and CL-poor (dark) regions occurs, as seen from the Liss Rhod PE membrane signal. Alexa Fluor 488 shows no distinct membrane accumulation in the absence of pTiO_2_ at either temperature. In contrast, localization occurs preferentially at CL-rich red regions upon pTiO_2_ addition in phase-separated GUVs. (D) QCM-d profile of the interaction of qPDMAEMA with supported lipid bilayers composed of DPPC:DOPC and DPPC:DOPC:CL (12.5 mol % CL), demonstrating that CL strongly promoted qPDMAEMA binding. Measurements were performed in Tris buffer at 25 °C in duplicate. (E) QCM-d profile of POPC:POPG (75:25 mol %) interaction with Alexa Fluor 488 (7 μM) in Tris buffer in the presence and absence of surface-bound qPDMAEMA, demonstrating that surface-bound qPDMAEMA strongly promoted surface binding of Alexa Fluor 488. Measurements were done at 25 °C in duplicate.

Cationic modification of photocatalytic NPs has attracted increasing attention in the last couple of years and was found to promote antimicrobial effects under illumination.^43^ Yet, the mechanistic origin of this boosting is rarely investigated beyond trivially noting that this is somehow related to electrostatic contrast between the net positively charged NPs and the net negatively charged bacteria. In one of few efforts to elucidate mechanistic aspects of the influence of surface coatings on the antimicrobial effects of photocatalytic NPs, Caselli et al. reported on photocatalytic TiO_2_ NPs coated with the antimicrobial peptide LL-37. In doing so, they systematically investigated effects of the surface coating on colloidal stability, NP binding to, and degradation of, lipid membrane mimics of bacteria and human cell membranes, as well as corresponding effects on bacteria and human cells.^18^ The peptide coating was found to improve colloidal stability, thereby increasing the effective surface area for ROS formation. In addition, the coating was found to be permeable to ROS and sufficiently stable under UV illumination to allow strongly enhanced membrane binding to bacteria-like model membranes. In contrast, binding to, and degradation of, human cell-like membranes were much less affected. Correspondingly, peptide coating strongly boosted antimicrobial effects against both Gram-negative and Gram-positive bacteria, at the same time as toxicity against human monocytes was minor. It was speculated that the effects observed were due to the coating driving proximity between the NPs and key components of the bacteria, including membrane lipids^18^ and lipopolysaccharides,^19^ allowing ROS generated at the NP surface to react with these components. Quantitatively, the ROS lifetime is very short (≈10^–9^, ≈10^–6^, and ≈10^–6^ s for ^•^OH, ^•^O_2_^–^, and ^1^O_2_, respectively^44^) corresponding to short diffusion lengths (≈1, ≈30, and ≈100 nm, respectively^23^), effectively limiting the distance reachable for ROS before they are consumed.

Despite providing valuable mechanistic insights into the enhanced antimicrobial performance of peptide-coated TiO_2_ NPs, the previous study left several critical questions unresolved. First, although peptide-coated NPs were shown to selectively oxidize bacteria-like model membranes, the causality between this selective oxidation and bacteria killing mechanism remained unresolved. Second, the finding that peptide coating did not impair ROS generation was limited to peptides of intermediate charge density. Coatings with substantially higher charge density, relevant for applications such as water disinfection,^28,29^ were not investigated, leaving an open question on how such coatings affect photocatalytic behavior and antimicrobial efficacy.^45^ Third, the previous investigations did not address whether the spatial selectivity of coated photocatalytic NPs extends beyond the basic bacteria-human cell distinction, leaving unexplored the potential for more nuanced, target-specific interactions. Since bacteria display inhomogeneities over the membrane surface in both lipid composition (e.g., cardiolipin)^46^ and lipopolysaccharides,^39^ it is possible that antimicrobial effects of photocatalytic NPs originate primarily from such ‘hot-spots’. Addressing all these remaining questions, the present study shows that (i) Oxidation promotes membrane defects that increase the permeability of bacteria-like GUVs and bacteria alike, (ii) high charge density coatings do not suppress ROS formation, (iii) pTiO_2_ binds preferentially in pole regions of *E. coli* bacteria, effects captured in bacteria-like GUVs and coupled to CL domain formation in these. These effects were observed for both the polymer-coated TiO_2_ NPs and the free polymer in solution, demonstrating that the localization level is unaffected by polymer immobilization to the NP surface. This suggests that polymer segment properties dictate the binding affinity of the coated NPs to the bacteria's membrane surface, consistent with the short Debye screening length (≈3.3 nm) under the present experimental conditions.

These findings, highlighting the importance of localized NP interactions with bacterial membrane domains, carry significant implications for the development and mechanistic evaluation of photocatalytic NPs as antimicrobial agents by demonstrating that membrane binding, defect formation, and permeabilization can occur preferentially in membrane domains whose compositions differ substantially from the average composition in the domain-free system (Figure 8). This, in turn, necessitates that model systems account for domain formation by identifying domains or regions along the membrane that constitute ‘hot-spots’ for permeabilization. While similar effects could potentially be obtained by conjugating the polymer to an organic photocatalyst (e.g., porphyrin derivatives), the presence of the nanoparticles in the present study offered the possibility to identify membrane-bound nanoparticles as ‘hot-spots’ for oxidative membrane destabilization, which would not be possible for such polymer conjugates. From an application perspective, the nanoparticles furthermore offer opportunities to pack these in processing units (e.g., fluidized beds) suitable for water flow-through under illumination, as well as relative ease of particle separation for replacement of the polymer coatings to increase the effective lifetime of the photocatalyst.^28^

**Figure 8:**
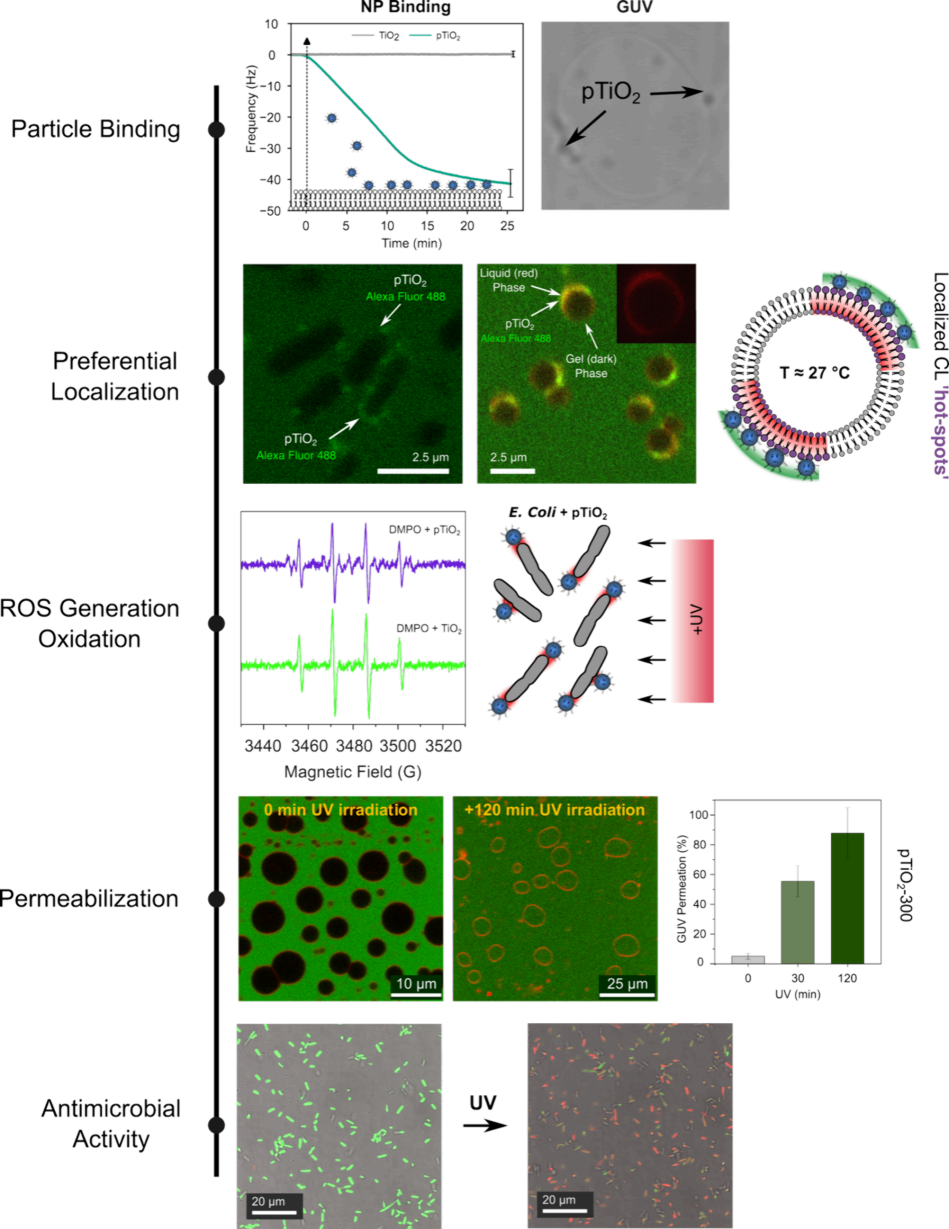
Illustration of key findings of the present study: Coating photocatalytic TiO_2_ NPs with qPDMAEMA led to strong interaction (adsorption) to bacteria-like membranes. It also did not affect the hydroxyl radical formation under UV illumination, which led to membrane destabilization and permeation. The antimicrobial activity of coated TiO_2_ NPs against Gram-negative *E. coli* was characterized by preferential binding and localized membrane permeability at the *E. coli* poles, where the rich presence of CL occurs. This preferential binding to CL-rich ‘hot-spots’ was also observed in two-phase GUV model membranes, characterized by CL-rich liquid and CL-poor gel domains.

While localized electrostatic interactions were used in the present study to target coated NPs for optimized oxidative damage to bacterial membranes by short-lived ROS, there are other means of localizing NPs to selective domains, including, e.g., aptamers, antibodies/antibody fragments, or small molecules (e.g., antibiotics or carbohydrates).^11,47^ Also here, the uneven distribution of recognition moieties (or their binding partner) along the bacterial membrane necessitates that model systems for studies of membrane interactions capture this uneven distribution.

Finally, it should be noted that domain formation in bacterial membranes is analogous to raft formation in eukaryotic cell membranes, characterized by colocalization of some lipids (e.g., cardiolipin) and scaffold proteins such as flotillins^48^ to enable efficient cellular processes such as signal transduction, protein secretion and folding, as well as cell division.^49^ As such, finding means to localize membrane perturbations by coated photocatalytic NPs may offer opportunities for selectively disturbing such processes, either as a research tool or in the search for new strategies for combatting challenging bacteria and bacterial infections.

## Conclusions

The present study demonstrates the mechanistic basis of enhanced antimicrobial activity of polymer-coated TiO_2_ NPs. Functionalization with cationic qPDMAEMA not only provided colloidal stability but also strengthened particle–membrane interactions and promoted selective binding to anionic bacteria-like phospholipid membranes. ROS generation was not suppressed by the polymer coating. Instead, the proximity of ROS sources to the membrane surface led to significantly higher rates of lipid oxidation, which facilitated membrane destabilization and permeation under UV exposure. Nanoparticle binding and photocatalytic effects were both found to occur preferentially at ‘hot-spots’ along the membrane surface, effects demonstrated to be governed by cardiolipin-rich domains. These findings demonstrate the importance of domains for membrane destabilization by photocatalytic nanoparticles, which need to be considered in the design of surface-functionalized nanoparticles with controlled selectivity and enhanced antimicrobial action (Figure 8). While the results demonstrate that pTiO_2_ NPs display potent (localized) membrane destabilization and antimicrobial effects, additional work is needed to elucidate if and how the system investigated lends itself to application in water disinfection. First, only a relatively small number of bacteria are imaged at a time in the LIVE/DEAD assay, and hence it is insufficient to determine if the bacterial count suppression by pTiO_2_ NPs meets the need for application purposes in water disinfection (at least a log 3–4 reduction, ideally more). Such biological assessment by *in vitro* killing or other macroscopic methods needs to be combined with other aspects on the practical implementation of qPDMAEMA-coated TiO_2_ NPs in water purification, such as reactor design, as well as potential photocatalyst doping to (i) allow visible light to be harvested and (ii) reduce electron–hole recombination for improved photocatalytic disinfection.

## Experimental Section

### Materials

TiO_2_ NPs (anatase) were obtained from PlasmaChem GmbH (Germany). Poly­(2-(dimethylamino)­ethyl methacrylate) methyl chloride quaternary salt (qPDMAEMA, *M*_w_: 8500 g/mol, CAS No.: 26161-33-1) was purchased from Sigma-Aldrich. The chemical structure of the polymer was confirmed by NMR (Figure S3).^34–36^ POPC (1-palmitoyl-2-oleoyl-*sn*-glycero-3-phosphocholine), POPG [1-palmitoyl-2-oleoyl-*sn*-glycero-3-phospho-(1’-rac-glycerol)], DPPC [1,2-dipalmitoyl-*sn*-glycero-3-phosphocholine], DOPC [1,2-dioleoyl-*sn*-glycero-3-phosphocholine], 18:1 cardiolipin [1’,3′-bis­(1,2-dioleoyl-*sn*-glycero-3-phospho)-glycerol (sodium salt)] (CL), and Liss Rhod PE [1,2-dioleoyl-*sn*-glycero-3-phosphoethanolamine-*N*-(lissamine rhodamine B sulfonyl) (ammonium salt)] were all of >99% purity and obtained from Avanti Polar Lipids (Alabaster, USA). Alexa Fluor 488 [Alexa Fluor 488 NHS Ester (Succinimidyl Ester)] and C_11_-BODIPY (581/591) [4,4-difluoro-5-(4-phenyl- 1,3-butadienyl)-4-bora-3a,4a-diaza-s-indacene-3-undecanoic acid] fluorophores were obtained from Thermo Fisher Scientific (USA). d-Mannitol (>98%) and superoxide dismutase (SOD) bovine (recombinant, ≥ 90%) were obtained from Sigma-Aldrich. Chloroform was obtained from VWR and 5,5-dimethyl-1-pyrroline-*N*-oxide (DMPO) radical (≥98%) from Cayman Chemicals. For acetate buffer (10 mM, pH 3.4), glacial acetic acid (99.9%), and sodium acetate (≥99.5%) were obtained from VWR and Sigma-Aldrich, respectively. Tris-HCl buffer (10 mM, pH 7.4 and 9.4) was prepared from Tris base (≥99.9%), obtained from Amersham Life Science (Buckinghamshire, UK). Analytical grades of sodium hydroxide (98–100.5%, pellets), calcium chloride (>99.5%), D_2_O (>99.9%), hydrochloric acid (37%), and methanol (≥99.9%) were obtained from Sigma-Aldrich. Ultrapure Milli-Q water (MQ water, 18.2 MΩ·cm) was used for all experiments. Unless otherwise stated, all experiments were conducted in 10 mM Tris buffer (pH 7.4), and error bars refer to standard errors from triplicate measurements.

### Surface Modification of TiO_2_ NPs

Initially, bare TiO_2_ dispersions (50 mg/L) were prepared at different pH values using acetate (pH 3.4) and Tris (pH 7.4, 9.4) buffers for initial characterization. Then, a series of qPDMAEMA-coated TiO_2_ (pTiO_2_) dispersions with fixed TiO_2_ and varying qPDMAEMA concentrations (mg polymer/g particles) were prepared to identify the lowest polymer-to-TiO_2_ ratio that gives rise to well-dispersed particles, thereby minimizing interference from free polymer in solution. For this, different volumes of qPDMAEMA were mixed with a given volume of bare TiO_2_ NPs at pH 11.0 and immediately subjected to 1 min vortex followed by 30 min tip sonication using an UP50H tip sonicator (Hielscher Ultrasonics GmbH, Germany). Due to the strong positive charge of qPDMAEMA and the titratable negative charge of TiO_2_ NPs, the resulting attractive electrostatic interaction promotes their coaggregation into hybrid nanoparticles consisting of multiple TiO_2_ NPs embedded into a qPDMAEMA matrix, and such particles are stable over at least 13 days. After that, Tris buffer (10 mM, pH 7.4) was added to obtain 50 mg/L TiO_2_.

### Light Scattering

Dynamic (DLS) and electrophoretic (ELS) light scattering were used to measure the particle size and electrophoretic mobilities of bare TiO_2_ and pTiO_2_ (Zetasizer Nano ZS, Malvern Panalytical Ltd., UK). Electrophoretic mobilities were converted to zeta potential values using the Smoluchowski equation, while backscattering at 173° was used to obtain number-average particle diameters. All measurements were performed in triplicate at 25 °C.

### Cryogenic Transmission Electron Microscopy (Cryo-TEM)

Experiments were conducted using a JEM-2200FS microscope (JEOL, Tokyo, Japan), equipped with TemCam-F416 camera (TVIPS). Aliquots (4 μL) of bare TiO_2_ or pTiO_2_ (50 mg/L) were applied onto glow-discharged Lacey Formvar grids (Ted Pella), blotted, and vitrified by rapid plunging into liquid ethane (−180 °C) under a controlled environment (relative humidity >90%, 21 °C). The grids were then stored in liquid nitrogen (−196 °C) and transferred to the cryo-transfer tomography holder (Fischione Model 2550) immediately prior to imaging. Images were analyzed using ImageJ (NIH, Bethesda, USA).

### Nuclear Magnetic Resonance Spectroscopy (NMR)

Liquid-state ^1^H NMR spectra for free qPDMAEMA and pTiO_2_ samples were recorded by using a Bruker Avance Neo 800 MHz spectrometer (Bruker BioSpin GmbH, Rheinstetten, Germany). Samples were prepared in Tris buffer. Due to line broadening of particle-bound polymer signals, the latter are strongly attenuated, and signals observed primarily originate from polymer molecules in solution. By comparing the NMR peak intensities of qPDMAEMA in solutions with and without TiO_2_, the fraction of free (nonadsorbed) polymer in each pTiO_2_ sample could be determined. Specifically, spectra of qPDMAEMA samples at 50 and 15 mg/L were compared with those of two pTiO_2_ samples containing a fixed TiO_2_ concentration (50 mg/L) and polymer loadings of 50 and 15 mg/L, corresponding to polymer-to-TiO_2_ ratios of 1000 mg/g and 300 mg/g, respectively. NMR spectra were acquired and processed by using TopSpin 4.5.0 (Bruker BioSpin GmbH, Rheinstetten, Germany).

### Electron Paramagnetic Resonance Spectroscopy (EPR)

To substantiate ROS identification and quantification, EPR spectra were recorded using a Bruker EMX Micro CW-EPR spectrometer (Bruker, USA), equipped with an ER 4119HS resonator. The microwave frequency was 9.766 GHz and the modulation frequency was 100 kHz. Final samples contained 240 mg/L particles and 40 mM DMPO as a spin-trapping agent.^50^ Samples were exposed to UV irradiation for 10 min, after which 200 μL aliquots were transferred to an EPR flat cell for measurement. Spectral simulations were carried out using EasySpin 6.0.0.

### Vesicle Preparation

Small aliquots of POPC and POPG in chloroform (10 mg/mL) were mixed in a dark and clean glass vial to a final POPC:POPG composition of 75:25 mol %. The mixture was then dried under N_2_ flow during gentle shaking, after which the dark vial was left in a vacuum desiccator for 2 h to evaporate the remaining solvent. The resulting dry lipid film was hydrated and dispersed in MQ water to a final lipid concentration of 0.1 g/L (or stored at −20 °C under N_2_ until further use to prevent oxidation). The solution was then subjected to 8 cycles of bath sonication (60 s) followed by vortexing (30 s). To obtain small unilamellar vesicles (SUVs) for bilayer formation (QCM-d measurements), the solution was further subjected to four 5 min cycles of tip sonication, separated by 2 min cooling periods using UP50H tip sonicator (Hielscher Ultrasonics GmbH, Germany). For the formation of C_11_-BODIPY-labeled vesicles (for the C_11_-BODIPY assay), dry lipid films were prepared similarly, with 0.5 mol % C_11_-BODIPY added from 10 mg/mL solution (in methanol) prior to the N_2_ drying step. The dry lipid film was hydrated with Tris buffer to reach a final lipid concentration of 2.0 mg/mL. The turbid lipid solution was then subjected to 1 min bath sonication followed by 31 repeated extrusions through 100 nm polycarbonate filters using LipoFast mini extruder (Avestin, Ottawa, Canada). The extrusion resulted in a clear solution, with vesicles characterized by a hydrodynamic radius of 106 ± 1 nm and a polydispersity index of 0.04.

### Quartz Crystal Microbalance with Dissipation (QCM-d)

A QSense analyzer (Biolin Scientific, Sweden), equipped with peristaltic pump and using a flow rate of 0.1 mL/min, was employed for QCM-d monitoring using SiO_2_ sensors (QSense QSX 303 SiO_2_, 4.95 ± 0.05 MHz, 14 mm diameter, 0.3 mm thickness, 17.7 ng/cm^2^ mass sensitivity). Initially, SiO_2_ sensors were cleaned by 10 min immersion (under bath sonication) in 2% Hellmanex, followed by MQ water, and then in ethanol. After drying under a stream of N_2_, the sensors were plasma cleaned for 5 min using model PDC-32G (Harrick Plasma, USA). After setting the water baseline, POPC:POPG bilayer deposition was achieved by continuous injection of SUVs (0.1 g/L), dispersed in MQ water. To remove excess vesicles, the bilayer was rinsed with MQ water for 5 min, followed by rinsing with Tris buffer for 10 min. The bilayer deposition consistently resulted in frequency and dissipation changes of −23.5 ± 0.9 (Hz) and 0.25 ± 0.02 (10^–6^), respectively, relative to MQ water baseline, in good agreement with previous observations.^51,52^ Different sets of experiments were then conducted to probe bilayer interactions with the various components, including free qPDMAEMA, bare TiO_2_ NPs, and pTiO_2_. For free qPDMAEMA, solutions (1–100 mg/L) were injected stepwise, followed by rinsing with Tris buffer for 60 min to remove any free polymer in solution. The binding of bare TiO_2_ NPs or pTiO_2_ to the deposited membrane was monitored similarly. After bilayer deposition, bare TiO_2_ or pTiO_2_ NPs were injected at a concentration of 20 mg/L for 10 min, followed by 10 min rinsing with Tris buffer to remove any free particles in solution. Subsequently, bilayers were irradiated by UV (UVP 3UV lamp, 254 nm, 8 mW, Analytik Jena, USA) for 120 min at 5 cm separation distance, using UV-transparent sapphire window modules (Biolin Scientific, Sweden). After 120 min, Tris buffer was injected to assess the reversibility. All measurements were performed at 25 °C in duplicate.

### C_11_-BODIPY Assay

C_11_-BODIPY is a hydrophobic fluorescent dye which localizes within the hydrophobic interior of the lipid bilayers.^53^ The oxidation of C_11_-BODIPY by ROS induces a shift in fluorescence from red (594 nm) to green (520 nm) which can be conveniently monitored by fluorescence spectroscopy.^54,55^ Fluorescence spectra were recorded for the C_11_-BODIPY-labeled vesicles in the absence and presence of particles using a Cary Eclipse fluorescence spectrophotometer (Agilent Technologies, USA). For this, C_11_-BODIPY-labeled vesicles and particles were mixed in Tris buffer to give final concentrations of 0.5 g/L vesicles and 20 mg/L particles. Equivalent particle-free samples were prepared as controls. Fluorescence spectra were recorded every 2 min with λ_ex_ of 485 nm and λ_em_ of 500–700 nm. The extent of oxidation was obtained by monitoring changes in green (520 nm) and red (594 nm) fluorescence. For each sample, the polarization index (PI) was calculated from the spectra as the ratio of oxidized (green) to total fluorescence as follows:

$$PI=\frac{I_{520}-I_{594}}{I_{520}+I_{594}}$$

PI values were related to the extent of oxidation for each time point by interpolation between the maximum and minimum PI values (−0.5 for 0% oxidation and 0.9 for 100% oxidation). Oxidation rates were obtained from time-oxidation graphs as slopes of initial linear fits for the first 5–10 min of UV exposure. To assess species contributing to C_11_-BODIPY oxidation, ROS scavengers were introduced in a separate set of experiments, using d-mannitol (·OH scavenger)^56^ and SOD (·O_2_^–^ scavenger)^57^ at final concentrations of 500 mM and 50 U/mL, respectively.

### Giant Unilamellar Vesicles (GUVs)

Individual POPC:POPG (75:25 mol %) and DPPC:DOPC:CL (43.5:43.5:12.5 mol %) lipid mixtures were labeled with 0.5 mol % red-fluorescing Liss Rhod PE. Similarly, a lipid mixture of POPC:POPG (75:25 mol %) was labeled with 0.5 mol % C_11_-BODIPY. The three lipid mixtures were then diluted in methanol:chloroform (1:9 volume ratio) solution to a final lipid concentration of 0.2 mg/mL. The GUVs were prepared by electroformation as described before.^58^ In short, a small aliquot (5–7 μL) from a given lipid mixture was deposited on the conductive side of an indium tin oxide (ITO)-coated coverslips (30–60 Ohms/Sq, Sigma-Aldrich), which was left to dry overnight in a vacuum chamber. Lipid-coated coverslips were affixed on the self-adhesive (bottom) side of an open-ended bottomless channel slide (sticky-Slide VI 0.4, Ibidi Inc., USA). This setup allows simultaneous addition of materials and real-time imaging of resulting effects. A noncoated ITO coverslip was mounted on the top surface of the channel, such that the two conductive sides face one another (hence, with the solution volume in between). The two ITOs were connected to electrical wiring (mounted to conductive side of the ITO glass) to enable electrical contact with electrodes of the frequency generator. The lipid film on the ITO glass was then hydrated with Tris buffer, and the sample was connected to a frequency generator for 3 h, where the generated AC electric field (10 V, 325 Hz) gave rise to a population of polydisperse GUVs.

### Confocal Microscopy

After electroformation, GUVs samples were observed using a confocal laser scanning microscope (Leica SP5, Leica Microsystems GmbH, Germany) operated in the inverted mode (D6000I) and equipped with temperature-controlled chamber with an accuracy of ±0.2 °C. Imaging was performed using an HC PL APO 100× oil immersion objective. Images were acquired at 1024 × 1024 pixels with a pinhole size of 1 Airy unit. Fluorescence was excited using the Argon (488 nm) and HeNe (543 nm, 633 nm) laser lines. The C_11_-BODIPY-labeled GUVs were imaged, before and after 2 h of UV illumination, to detect the normal (510–530 nm) and oxidized states (580–600 nm) of the C_11_-BODIPY. Alexa Fluor 488 was detected at 500–550 nm, and Liss Rhod PE at 560–620 nm. The DPPC:DOPC:CL GUVs were imaged at 45 °C (single-phase GUVs) and, after cooling, at 27 °C (two-phase GUVs).

### Live/Dead Bacterial Assay

Bacteria (*E. coli* ATCC 25922) were grown to stationary phase in 35 mL Lennox broth (LB Broth, Sigma-Aldrich, USA) overnight at room temperature. Bacteria were then collected by two rounds of centrifugation (10 min, 10,000*g*) and resuspended in Tris buffer. Samples were prepared in quartz cuvettes with a final particle (TiO_2_ or pTiO_2_) concentration of 5 mg/L and an OD600 of 0.6, corresponding to 6 × 10^8^ colony-forming units (CFU)/mL. The samples were then incubated for 1 h at room temperature under UV illumination (UVP 3UV Lamp, 254 nm, 8 mW, Analytik Jena, USA) with a cuvette-lamp separation distance of 6 cm. Particle-free (control) samples, as well as those with free qPDMAEMA (1.5 mg/L) were prepared similarly. In addition, equivalent samples were prepared and incubated in darkness for 1 h. After incubation, bacteria were stained using the LIVE/DEAD BacLight Bacterial Viability Kit (Thermo Fisher Scientific Inc., Waltham, USA) by mixing 200 μL of a given sample with 0.5 μL of a 1:1 (v/v) mixture of SYTO 9 (λ_ex_/λ_em_ 480/500 nm) and propidium iodide (λ_ex_/λ_em_ 490/635 nm). After 10 min staining, each sample was imaged by confocal microscopy, where 20 wide-field images were collected. The proportion of dead bacteria was quantitatively analyzed using ImageJ (NIH, Bethesda, USA). LIVE/DEAD quantification was performed using independently prepared samples and multiple imaging regions per condition to ensure reproducibility, with variability reported accordingly. While this assay provides reliable comparative assessment of membrane damage, it primarily reflects membrane integrity within the analyzed bacterial population rather than large-scale bacterial count suppression.

### Fluorescent Mapping of *E. coli*-qPDMAEMA Interaction

*E. coli* bacteria (ATCC 25922), grown to a stationary phase, were collected by two rounds of centrifugation (10 min, 10,000*g*) and resuspended in Tris buffer. Samples were prepared to a final particle (TiO_2_ or pTiO_2_) concentration of 10 mg/L and an OD600 value of 0.6. Samples were then incubated for 2 h (in the dark or under UV). Particle-free (control) samples as well as samples containing free qPDMAEMA (3 mg/L) were prepared similarly. After incubation, Alexa Fluor 488 was introduced into the sample, which was then imaged via confocal microscopy.

## Supplementary Material



## Notes

TEM imaging of bare TiO_2_; particle size and zeta potential for bare and qPDMAEMA-coated TiO_2_ particles after 13 days; ^1^H NMR analysis of qPDMAMEA for determination of optimal qPDMAEMA loading and saturation on TiO_2_; kinetics of ROS-induced oxidation by TiO_2_ particles under UV exposure; QCM-d profiles of POPC:POPG bilayer formation and subsequent UV treatment; QCM-d profiles of bilayer interaction with free qPDMAEMA, as well as bare and coated TiO_2_ particles; confocal microscopy images and intensity analysis of GUVs in the presence of bare TiO_2_ before and after UV exposure; LIVE/DEAD assay of *E. coli* in the presence of free qPDMAEMA; confocal microscopy imaging of domain formation and CL-mediated qPDMAEMA localization on DPPC:DOPC:CL GUVs (PDF)
