# Supplementary material for: Polymer-Coated Nanoarchitectonics of Titanium Dioxide Nanoparticles with Enhanced Antimicrobial Activity: Mechanistic Insights into Localized Particle–Membrane Interactions
Source: ACS Cent Sci. 2026 Jun 18;12(7):951–64. doi: 10.1021/acscentsci.6c00511 (PMC13397284; doi:10.1021/acscentsci.6c00511)
Supplement: Supplementary file 1 [file oc6c00511_si_001.pdf]

Supporting Information

Polymer-Coated Nanoarchitectonics of Titanium Dioxide Nanoparticles with Enhanced Antimicrobial Activity: Mechanistic Insights into Localized Particle-Membrane Interactions

Nizar B. Alsharif,[1,2,*] *Lucrezia Caselli,*[1,3,4] *Anders Thapper,*[5] *Emma Sparr,*[1,2] *Martin Malmsten*[1,6]

[1]Department of Chemistry, Lund University, SE-22100 Lund, Sweden

[2]Wallenberg Initiative Materials Science for Sustainability, Department of Chemistry, Lund University, SE-22100 Lund, Sweden

[3]Department of Chemistry, University of Florence, Via della Lastruccia 3, 50019 Sesto Fiorentino, Florence, Italy.

[4]Center for Colloid and Surface Science (CSGI), Via della Lastruccia 3, 50019 Sesto Fiorentino, Florence, Italy.

[5]Department of Chemistry - Ångström, Uppsala University, SE-75120 Uppsala, Sweden

[6]Department of Pharmacy, University of Copenhagen, Copenhagen DK-2100, Denmark

***Corresponding author.** Email: nizar.alsharif@chem.lu.se

Table of Contents

Figure S1.	TEM image of bare TiO_2 NPs	S2
Figure S2.	Particle size and zeta potential of $pTiO_2$ at 0 and 13 days	S3
Figure S3.	Liquid-state ^1H NMR spectra of unbound qPDMAEMA	S4
Figure S4.	UV-induced C_{11}-BODIPY oxidation in lipid membranes	S5
Figure S5.	QCM-d profile of POPC:POPG supported bilayer formation	S6
Figure S6.	QCM-d profile of bilayer interaction with free qPDMAEMA	S7
Figure S7.	QCM-d profile of UV treatment of bilayer in the absence of NPs	S8
Figure S8.	QCM-d profiles of bilayer interaction with bare and coated TiO_2 NPs	S9
Figure S9.	Confocal analysis of bare TiO_2-induced permeabilization in GUVs	S10
Figure S10.	LIVE/DEAD confocal microscopy of qPDMAEMA-treated *E. coli*	S11
Figure S11.	CL-mediated qPDMAEMA localization in phase-separated GUVs	S12

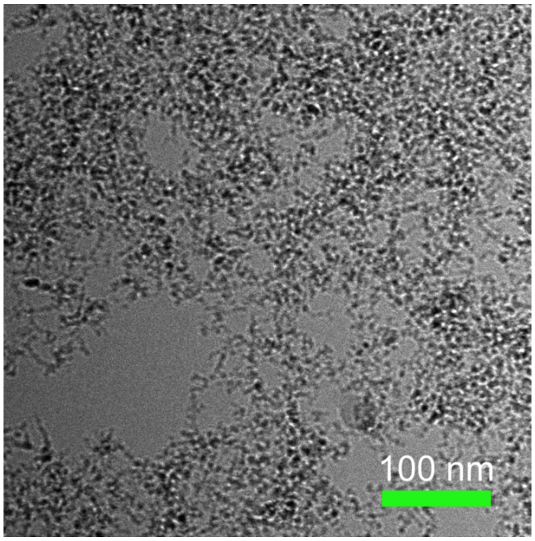

Figure S1. Representative cryoTEM image of bare TiO_2 NPs (50 mg/L) in Tris buffer.

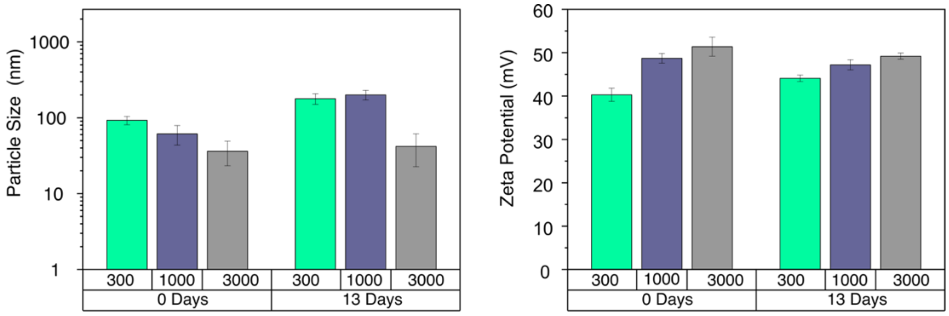

Figure S2. Hydrodynamic diameters and zeta potential values of $pTiO_2$ (50 mg/L TiO_2) in Tris buffer for selected qPDMAEMA concentrations upon sample preparation (0 days) and after 13 days. Measurements were performed in triplicate at 25 °C.

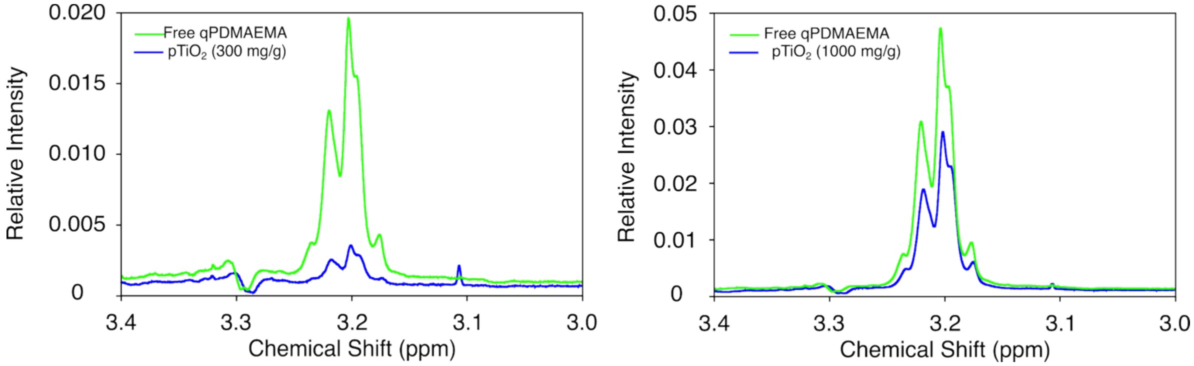

Figure S3. Liquid-state ^{1}H NMR spectra of unbound qPDMAEMA in $pTiO_2$ samples with fixed TiO_2 (50 mg/L) and varying qPDMAEMA concentrations (blue), compared with reference polymer solutions containing equivalent qPDMAEMA concentrations (green). The ~3.2 ppm peak corresponds to quaternary ammonium protons [–$N^+(CH_3)_3$]. Spectra were recorded in Tris buffer to evaluate polymer adsorption and surface saturation through attenuation of the –$N^+(CH_3)_3$ peak.

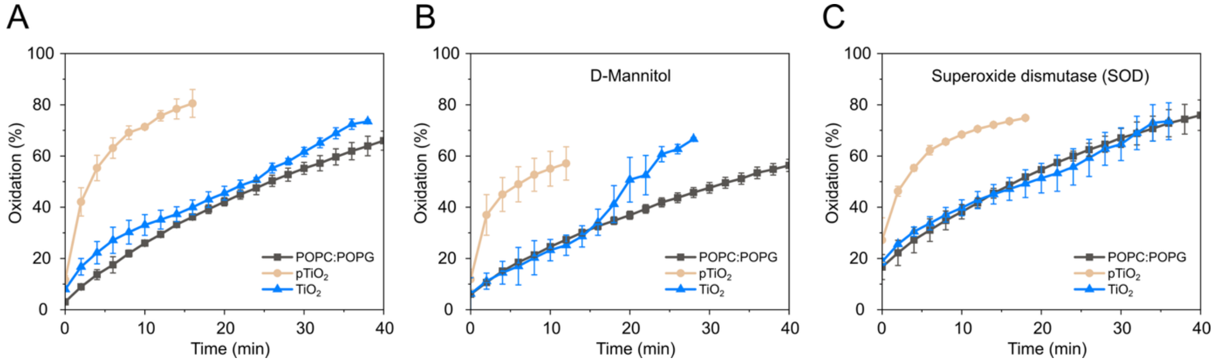

Figure S4. Results of the C_{11}-BODIPY assay. **(A)** Oxidation levels of C_{11}-BODIPY in the absence of NPs (POPC:POPG), as well as in the presence of bare TiO_2 or $pTiO_2$ NPs (both 20 mg/L) under UV irradiation. Oxidation levels in the presence of **(B)** 500 mM D-mannitol (•OH scavenger), and **(C)** 50 U/mL SOD (•O_2^- scavenger). Measurements are performed at 25 °C in triplicate in Tris buffer.

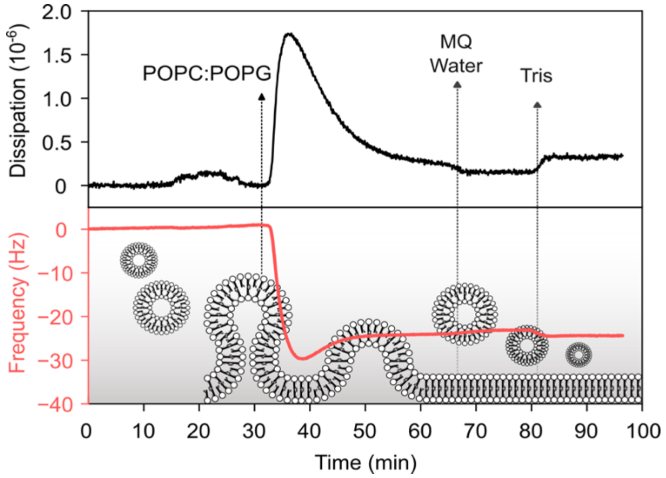

Figure S5. Representative QCM-d profile of the POPC:POPG SUV deposition and subsequent bilayer formation. Shown are frequency and dissipation changes after addition of 0.1 g/L (POPC:POPG 75:25 mol%), followed by subsequent rinsing with MQ water and Tris buffer. Shown also a graphic representation of the process monitored.

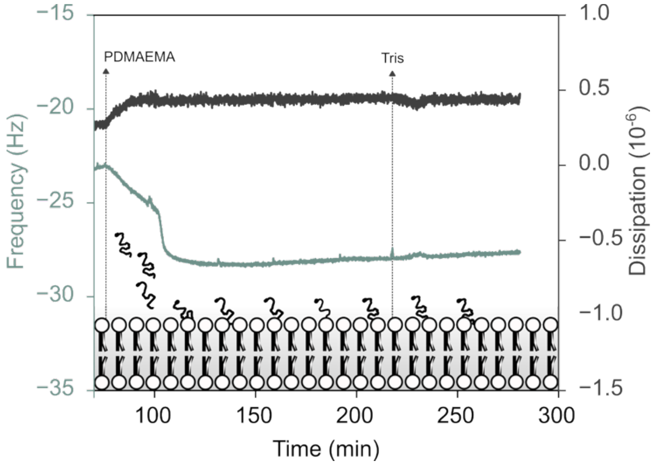

Figure S6. QCM-d profile showing frequency and dissipation changes during bilayer interaction with free qPDMAEMA (1-100 mg/L), where the polymer was injected after bilayer deposition followed by rinsing with Tris buffer.

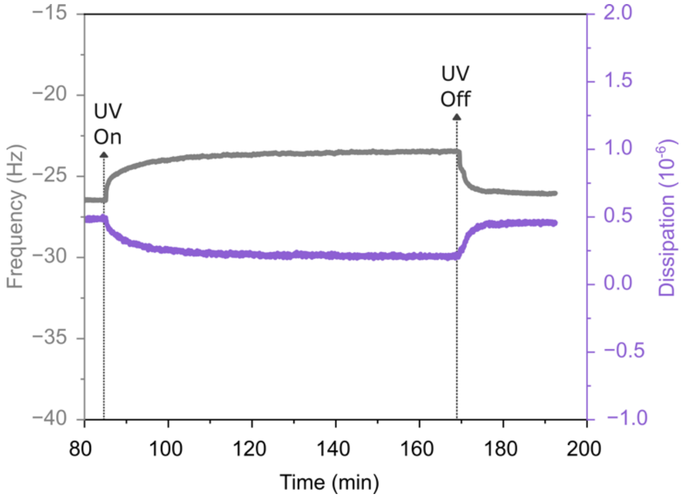

Figure S7. QCM-d profile of POPC:POPG bilayer, subjected to UV irradiation in the absence of photocatalytic NPs. The bilayer was rinsed with Tris buffer both prior to and following UV treatment. Bilayer deposition profile (**Figure S5**) is not shown.

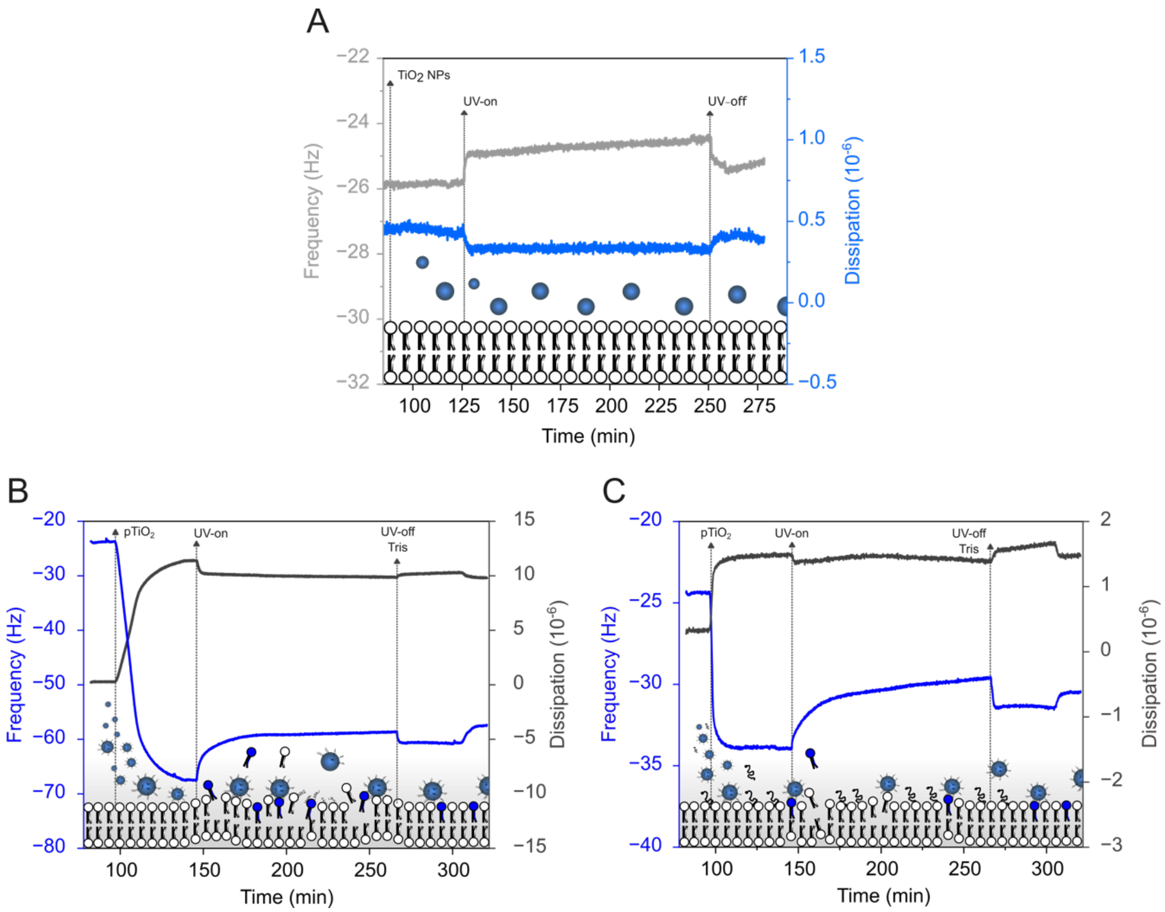

Figure S8. Full QCM-d profile showing frequency and dissipation changes during bilayer interaction with **(A)** bare TiO_2 NPs (20 mg/L), **(B)** pTiO_2 (20 mg/L TiO_2 NPs, 300 mg/g), and **(C)** pTiO_2 (20 mg/L TiO_2 NPs, 1000 mg/g). NPs/polymer was injected after bilayer deposition followed by rinsing with Tris buffer, which was stopped prior to UV illumination.

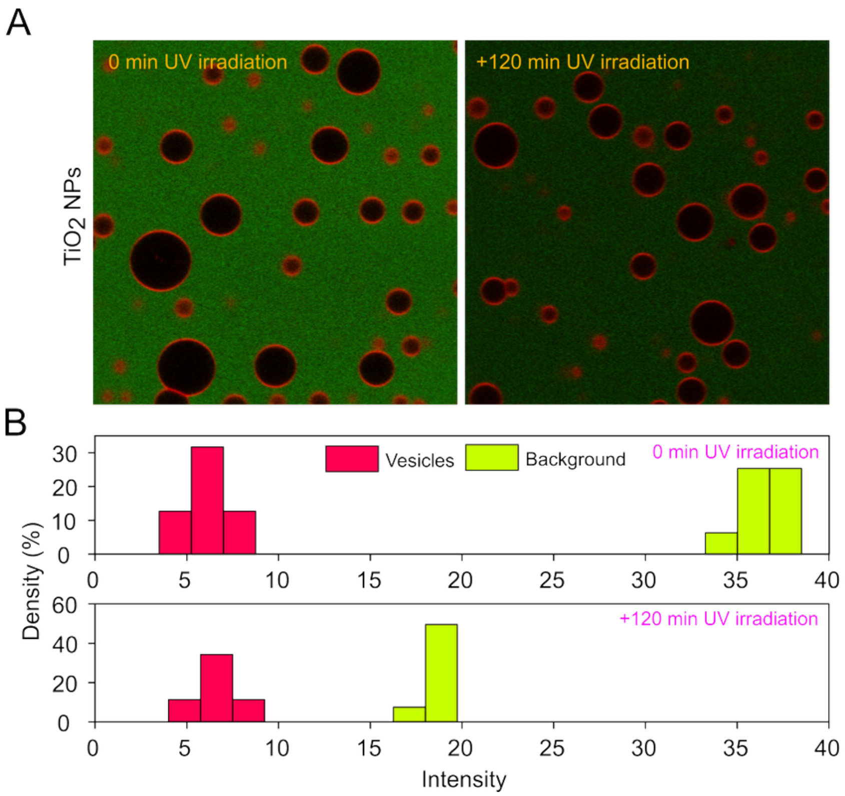

Figure S9. **(A)** Overlaid confocal microscopy images (red/green channels) of Liss Rhod PE-labeled POPC:POPG GUVs, dispersed in Alexa Fluor 488 and incubated with bare TiO_2 NPs (1 mg/L) before (left) and after (right) UV irradiation (120 min). **(B)** Fluorescence intensity analysis of Alexa Fluor 488 inside (red bars) and outside (green bars) vesicles.

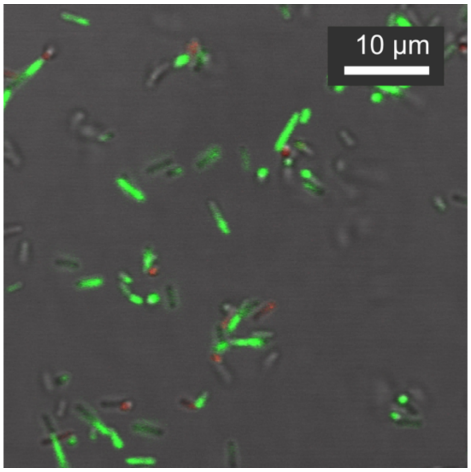

Figure S10. Representative confocal microscopy images for LIVE/DEAD assay for *E. coli* (6 × 10^8 CFU/mL) incubated with qPDMAEMA (1.5 mg/L) for 1 h in darkness. Background antimicrobial activity of free qPDMAEMA was 14 ± 6% dead bacteria.

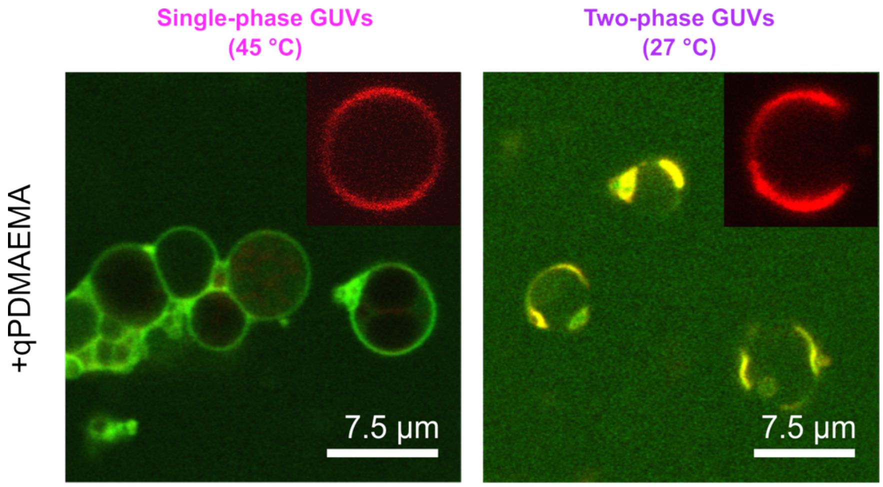

Figure S11. Confocal microscopy images of CL-mediated qPDMAEMA (5 mg/L) localization onto DPPC:DOPC:CL GUVs in Alexa Fluor 488-containing Tris buffer. As shown, qPDMAEMA bound uniformly around single-phase GUVs at 45 °C, as inferred from the binding of negatively charged Alexa Fluor 488 (green) to polymer-coated GUVs. When formed 45 °C and cooled to 25 °C, GUVs phase separate into CL/DOPC-rich (red) and DPPC-rich (dark) regions, where addition of qPDMAEMA result in preferential localization to CL/DOPC-rich red regions.
